# Quality of life and sexual function after high-dose or conventional chemotherapy for high-risk breast cancer

**DOI:** 10.1038/sj.bjc.6603454

**Published:** 2006-12-12

**Authors:** K M Malinovszky, A Gould, E Foster, D Cameron, A Humphreys, J Crown, R C F Leonard

**Affiliations:** 1 South West Wales Cancer Institute, Singleton Hospital, Swansea University, Swansea SA2 8QA, UK; 2 Quantics Consulting Limited, Kippilaw Mains, Melrose TD6 9HF, UK; 3 NHS Scotland, Information and Statistics Division, 1st Floor, Gyle Square, South Gyle, Edinburgh EH12 9EB, UK; 4 Edinburgh University, Western General Hospital, Crewe Road South, Edinburgh EH4 2XU, UK; 5 James Cook University Hospital, Middlesbrough TS4 3BW, UK; 6 St Vincents University Hospital, Elm Park, Dublin 4, Republic of Ireland; 7 Hammersmith Hospitals NHS Trust and Imperial College, Du Cane Road, London, W12 0HS, UK

**Keywords:** sexual function, quality of life, chemotherapy, breast cancer

## Abstract

Three hundred and ninety women participated in the quality of life (QL) study of ACCOG1, a high-dose *vs* conventional adjuvant chemotherapy breast cancer trial, for patients with a high risk of relapse. Patients completed the European Organisation for Research and Treatment of Cancer QLQ-C30, questions on menopausal symptoms and the Sexual Activity Questionnaire. Pretreatment, 6,12, 24, 36, 48 and 60-month assessments were conducted. For the high dose group the median decrease in global QL at 6 months was significantly greater than in the conventional group. At 12 months, however, the median change had returned to 0 for both groups. Social functioning was also significantly lower in the high-dose group at 6 months, again returning to prebaseline levels for both groups after 12 months. The most persistent changes appear to be in the effect of treatment in both arms on sexual outcomes, reflected in problems with discomfort and pleasure. Both high-dose and conventional chemotherapy showed persisting negative effects on sexual health. This has not been previously reported as a long-term complication of high-dose chemotherapy. However, it did not have long-term affects on sexual habit, which appeared to return to pretreatment frequency and similar to that of conventional chemotherapy by about 12 months from treatment.

Breast cancer patients with four or more positive axillary lymph nodes have historically had a poor prognosis (Jatoi *et al*, 1999), even with conventional adjuvant chemotherapy (EBCTCG, 2005). A series of uncontrolled studies of high-dose chemotherapy in the 1990s, mainly using an induction/intensification strategy, in which patients received conventional chemotherapy followed by high-dose chemotherapy as a late intensification regimen, produced results that showed a substantial benefit for high-dose regimens (Antman *et al*, 1992; Peters *et al*, 1993). High-dose therapy soon became a widely used treatment option for women with bad-risk breast cancer, even without supporting evidence from randomised controlled trials.

The Anglo Celtic 1 trial was set up to examine the potential benefit, if any, of employing high-dose chemotherapy in the treatment of early stage bad-risk breast cancer based on the involvement of the axillary nodes at diagnosis. One of the prospective substudies within the Anglo-Celtic 1 trial was to examine the impact of late intensification high-dose chemotherapy on QL. The purpose of this report is to review the detailed analysis of patient QL. Ethical approval for all parts of the study was given from the MREC for Scotland.

## PATIENTS AND METHODS

Between February 1995 and June 1999, 605 patients with newly diagnosed breast cancer with four or more positive lymph nodes were randomly assigned to either conventional (*n*=298) or high-dose treatment (*n*=307). Both treatment arms initially received four cycles of doxorubicin 75 mg m^−2^. Patients in the conventional arm then received eight further cycles of CMF (cyclophosphamide (600 mg m^−2^), methotrexate (50 mg m^−2^) and 5-fluorouracil (600 mg m^−2^) while the high-dose group received a single cycle of intermediate-dose cyclophosphamide (4000 mg m^−2^) supported by filgrastim (300 *μ*g day^−1^) for up to 10 days followed by high-dose cyclophosphamide (6000 mg m^−2^) and thiotepa (800 mg m^−2^). Peripheral blood progenitor cells were harvested by leukapheresis after treatment with cyclophosphamide and filgrastim and re-infused after the high-dose cycle. A full report of the main study has been published (Leonard *et al*, 2004).

### Quality of Life

The two arms of the trial differed in two ways with regard to QL. In the conventional arm, therapy continued over a much longer period – 9 months *vs* 4 months in the high-dose arm. But in the high-dose arm treatment was more intense with the risk of severe short-term side effects. It was decided that QL would be compared initially at 6 months following the start of treatment. Those on the high-dose regimen would have completed their treatment, while patients in the conventional arm were still having treatment. It was planned that later assessments would give an indication of the global QL of all patients having completed their treatment and, importantly, as adjuvant chemotherapy regimens that include cyclophosphamide have been reported to induce high levels of premature menopause (Bines *et al*, 1996), the effects of these treatments on menopausal symptoms and sexual function. The main aims of the QL study were to assess and compare the two groups with respect to their reported:

1. global QL
2. fatigue
3. menopausal symptoms
4. sexual function.

### Instruments

#### QLQ-C30

The EORTC QLQ-C30 (Aaronsen *et al*, 1993) is a 30-item self-report questionnaire that incorporates functional scales (physical, role, emotional and social) a global QL scale, 3-symptom scales (fatigue, pain, nausea and vomiting) and a number of single item measures. Published data show its practicality, reliability and validity in a variety of patient groups (Osoba *et al*, 1994: Apolone *et al*, 1998). The QLQ-C30 has been used extensively in studies with breast cancer patients. The global QL and fatigue scales were considered of particular relevance to this study (Curt, 2000; Cella *et al*, 2004). Also, the ratio of cost to the patient (side effects) to the benefit (disease control/survival) is crucial to the evaluation of treatment worth. Assessment of global QL is derived from two items concerning overall health and global QL rated on a 7-point scale (very poor to excellent). Item scores are summed and linearly transformed (0–100). A higher score represents a better quality of life. The fatigue scale consists of three items (concerned with need for rest, weakness and tiredness), each scored on a 4-point scale (not at all to very much). Item scores are summed and linearly transformed (0–100). A high score represents a higher level of fatigue.

#### Menopausal symptoms

Menopausal symptoms are not adequately monitored by the QLQ-C30, therefore additional items were required. The items chosen were derived following consultation with clinicians involved in the ABC trial – following a review of the literature and of the women's health questionnaire. The items selected concerned the experience of termination of menstruation, night sweats, day sweats, vaginal dryness, weight gain, and aches and pains. These were adapted to conform to the response format of the EORTC questionnaire.

#### Sexual activity questionnaire

Sexual function is a difficult dimension of QL to assess by self-report questionnaire and responses are likely to reflect affective and interpersonal factors as well as their psychological status. Because of the importance attached to improving understanding of sexual outcomes, it was decided to incorporate the Sexual Activity Questionnaire (SAQ) into this study. The SAQ (Thirlaway *et al*, 1996) was developed for another study (Fallowfield *et al*, 2001) and is designed to measure sexual functioning in terms of activity, pleasure and discomfort.

#### Assessment schedule

Quality of life measures were assessed before randomisation, at 6 months, 1 year and annually thereafter up to 5 years. Questionnaires were administered by research nurses at the recruiting centres. Follow-up for QL ceased if a breast cancer recurrence was diagnosed.

### Study population

Eligible patients were patients who were entered into the high dose *vs* conventional adjuvant chemotherapy breast cancer trial (ACCOG1) trial were able and willing to give informed consent to participate in the QL study and able to read and complete the self-report QL questionnaire. Three hundred and ninety patients from 27 centres completed at least one QL questionnaire.

### Statistical methods

All statistical tests were two-sided. Tests were reported as significant if *P*<0.05. No formal adjustments were made for multiple testing but *P*-values were reported to allow judgement to be used in the interpretation of the results. Scores were compared between treatment groups using the Mann–Whitney test. Changes over time in scores were calculated per patient and hence tests were restricted to those patients responding at both of the time points on question. Proportions were compared using Fisher's exact test. Changes in proportions over time were compared using the McNemar test (to allow for the pairing of binary responses in an individual patient).

## RESULTS

### Patient characteristics

Demographic data and clinical characteristics of the two groups within the QL study were compared to those of all study patients (Table 1). Mean age of all patients in the QL study was 45 years for high dose and 46 for conventional therapy patients. At the start of the study, 67% of patients in the high-dose arm and 63% of patients on conventional therapy were known to be premenopausal, with 80 and 79% of all patients receiving tamoxifen, respectively.

### Response rate

Table 2 shows the numbers of QL forms returned at each time point, compared with the numbers expected (allowing for recurrences and deaths). At baseline, 77% of forms were returned, decreasing to 71% after 2 years and to 50% after 5 years.

### Completeness of forms

The EORTC QLQ-C30 items were very well completed up to 2 years, with at most two missing values per dimension in either treatment group, and at most four missing values per symptom.

The menopausal items 1–5 were generally well completed, with the exception of the premenopausal group at baseline where about 10% of values were missing. The remaining questions were less well completed at the early time points.

For the sexual activity questions, although those on page 1 were well completed (mainly less than 5% missing), many more responses were provided for the page 2 questions (relevant only to sexually active patients) than patients stating that they were sexually active. Analysis excluded patients who said they were not sexually active at any given time point.

### Primary end points

Note that the graphs include all patients who responded at each time point. Groups are not directly comparable in the graphs as the groups of patients who responded differ over time. To make statistical comparisons, we have to consider changes from baseline for those patients who responded at both baseline and the time in question.

### Global QL

**Figure 1:**
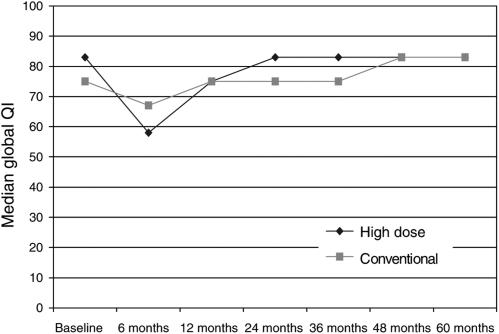
Global quality of life.

For the high-dose group, the median decrease in global QL at 6 months was significantly greater (*P*=0.02) than in the conventional group (Figure 1). At 12 months, the median change had returned to baseline levels in both groups.

### Fatigue

**Figure 2:**
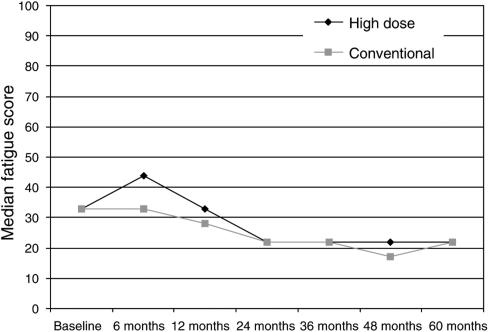
QLQ-30 fatigue score.

For the high-dose group, there was a median increase in fatigue at 6 months; median levels of fatigue were unchanged in the conventional group at this time point but not significantly different from the high-dose group (*P*=0.06) (Figure 2). At 12 months, the median fatigue scores had returned to baseline levels in both groups and continued to fall until 24 months. This lack of difference in fatigue in the conventional group at 6 months is surprising and may reflect a lack of sensitivity for picking up changes in fatigue in the QLQ C30.

### QLQ dimensions and symptoms

**Figure 3:**
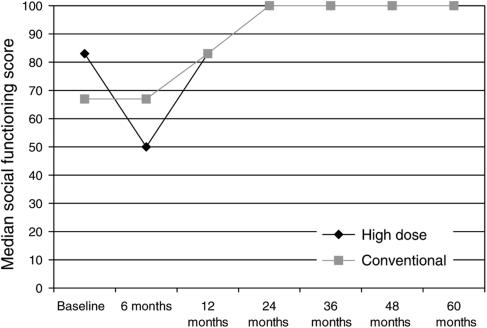
QLQ social functioning score.

The only dimension showing a significant difference between treatment groups in change from baseline was social functioning at 6 months (*P*<0.002), with patients in the high-dose treatment arm having lower social functioning scores than the conventional arm (Figure 3). Both of these scores returned to baseline levels at 12 months and improved on baseline scores (collected following diagnosis and surgery for breast cancer but before the start of chemotherapy) from 2 years onwards.

The only symptoms showing significant difference between treatment groups in change from baseline were appetite change (*P*<0.001) and constipation (*P*<0.001) at 6 months. High-dose patients suffered more appetite loss and less constipation than conventional patients at this point.

### Menopause

Of the women who were premenopausal before entry into the trial, 113 (86%) and 96 (79%) of patients in the high-dose and conventional groups, respectively, experienced loss of menses during follow-up. There was no significant difference between the groups.

### Menopausal symptoms

For patients in both groups, night sweats, daytime sweats, hot flushes and vaginal dryness increased over the first year and remained significantly above baseline over the 5-year follow-up (*P*<0.0001 at each time point). Aches and pains were increased at 6 months (*P*=0.0004), 48 months (*P*=0.006) and 60 months (*P*=0.002). Weight gain was significantly above baseline up to 2 years (*P*<0.0001), thereafter there was no significant difference from baseline. Menstrual problems did not change significantly over the period, having had a mean score of 1.3 at baseline (where 1=‘Not at all’ and 2=‘A little’). Patients' feelings about their menstrual state scored a mean of 5.4 at baseline (towards the relieved end of the scale of 1 to 7) and increased steadily up to a mean value of 5.9 at 5 years.

The only differences between the two treatment groups were for aches and pains and weight gain at 6 months (*P*<0.05). In both cases, the high-dose group saw smaller changes from baseline than the conventional group.

### Sexual activity

**Figure 4:**
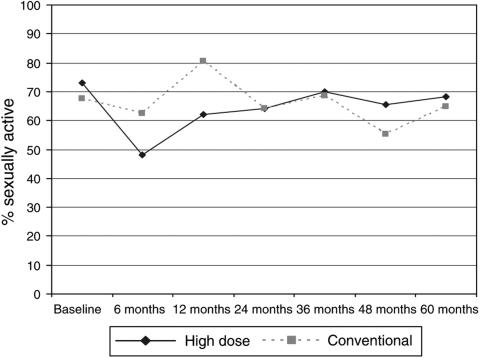
Proportion of women who were sexually active.

Data on sexual activity over 5 years are presented in Table 3. Of the patients who responded to the question ‘Do you engage in sexual activity with anyone at the moment?’, 108 out of 148 (73%) of high-dose patients and 92 out of 147 (68%) of conventionally treated patients were sexually active at baseline; the groups were not significantly different (*P*=0.4). For the high-dose group, sexual activity was significantly lower than baseline at 6 and 12 months (Figure 4, *P*⩽0.001, *P*=0.03), but returned to baseline levels thereafter. For the conventional group, there were no significant changes from baseline.

### Sexual outcomes

**Figure 5:**
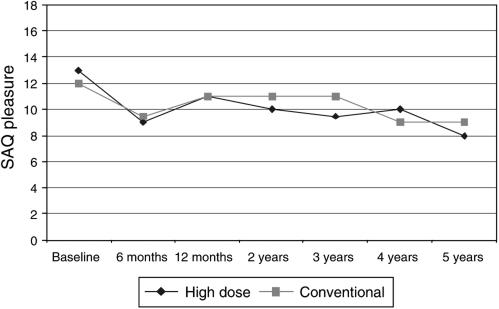
SAQ pleasure (high score=high pleasure).

For patients who responded to the questions on sexual pleasure, the median score was 13 at baseline (Figure 5); the groups were not significantly different (*P*=0.8). The changes from baseline were not significantly different between the treatment groups at 6 months (*P*=0.6) or 12 months (*P*=0.9). For both the groups, pleasure was significantly reduced from baseline at all time points up to 5 years. When patients were compared by menopausal status at trial entry, there were no significant differences in pleasure scores between the two treatment groups.

For patients who responded to the questions on discomfort, the median score was 0 at baseline; the groups were not significantly different (*P*=0.9). The changes from baseline were not significantly different between the treatment groups at 6 months (*P*=0.2) or at 12 months (*P*=0.6). For the patients as a whole, discomfort was significantly increased from baseline at all time points up to 5 years (*P*<0.001 at all time points). Again, there were no significant differences when patients were compared by menopausal status at trial entry.

Overall patients who responded to the question on frequency of sexual activity over the past month compared with ‘what is usual for you’, the median score at baseline was 1 ‘about the same’. The median score for the high-dose group was 1 at baseline and 0 ‘less than usual’ for the conventional group; the difference between the groups was not significant (*P*=0.4). The changes from baseline were not significantly different between the treatment groups at 6 months (*P*=0.5) or at 12 months (*P*=0.7). For the patients as a whole, frequency of sexual activity compared with usual was significantly reduced from baseline at 6 months (*P*<0.001) but was similar to baseline at all time points beyond that. At 6 months, there was a small difference in frequency between pre- and postmenopausal patients – a larger drop for premenopausal patients – this suggests that the treatment effect at 6 months is concentrated in the premenopausal patients. These differences disappear at 12 months and in subsequent years.

## DISCUSSION

The early data collection was probably adequate to draw some general conclusions about the impact on the QL in the early to medium term following highdose as compared to conventional chemotherapy. The important general conclusion is that although the median decrease in global QL in 6 months was greater in the high-dose chemotherapy group as compared to the conventional chemotherapy group, the changes had returned to normal and were equal to the conventional therapy group by 12 months. Over the same time period, there was also a trend to increased fatigue following high-dose chemotherapy but this did not reach significant levels. Deterioration in social functioning from baseline was significantly greater in the high-dose cancer group but again this returned to the same levels as conventional chemotherapy by 12 months, in both groups social functioning scores reached higher than baseline levels at 2 years. Lower social functioning scores at baseline were likely to reflect a recent diagnosis of cancer and breast cancer surgery.

The most persistent changes appear to be in the effect of treatment on menopausal symptoms and on sexual pleasure, also reflected in problems with discomfort. These details shown in Table 3 show that high-dose and conventional chemotherapy have persisting negative effects on sexual health that may last for several years following therapy for both pre- and postmenopausal women. Data on the effect of other hormone-related treatments on sexual discomfort were not collected and could therefore have influenced sexual outcomes in this study, both groups had similar levels of tamoxifen use (80% high dose; 79% conventional treatment). As over 60% of patients were premenopausal before chemotherapy, it is likely that some of these problems were associated with menopausal symptoms. In another study, chemotherapy-induced menopause has been shown to be related to greater changes in sexual functioning, although women who were menopausal before cancer chemotherapy have also reported changes in sexual function, of a lower order (Lindley *et al*, 1998).

To the best of our knowledge, changes in sexual pleasure and discomfort have not been previously reported as a long-term complication of high-dose chemotherapy. These changes, however, did not have long-term effects on sexual habit, which appeared to return to normal frequency and similar to that of conventional chemotherapy by about 12 months from treatment.

## CONCLUSIONS

Overall these results suggest that high-dose chemotherapy had temporary differentially more potent effects on several areas of QL and sexual activity in the first 6 months following treatment. These virtually return to baseline levels of function similar to conventional therapy after this time point. The one exception is sexual health, with sexual pleasure significantly reduced and discomfort increased for up to 5 years following both conventional and high-dose chemotherapy. These results should be reviewed within the context of the main study (Leonard *et al*, 2004) that reported no significant differences between the groups in 5-year relapse-free survival and overall survival. It would therefore seem that the increased early toxicities associated with the high-dose therapy are not balanced by improvements in early 5-year survival.

## Figures and Tables

**Table 1:** Demographic and clinical characteristics of QL patients

	**QL Study**				**Main study**	
	**High dose *N*=198**		**Conventional *N*=192**		***N*=605**	
Age mean, 95% CI	45	(22, 60)	46	(25, 63)	46	(2, 64)
						
Menopausal status	*N*	% of known	*N*	% of known	*N*	% of known
Premenopausal	132	67	121	63	400	66
Perimenopausal	23	12	19	10	62	10
Postmenopausal	42	21	51	27	138	23
Missing	1		1		5	
						
*Radiation to the breast*						
Yes	179	95	179	95	547	95
No	10	5	10	5	30	5
Missing	9		3		28	
						
*Radiation to the axilla*						
Yes	39	21	46	24	156	27
No	150	79	143	76	421	73
Missing	9		3		28	
						
*Tamoxifen*						
Yes	152	80	150	79	465	81
No	36	20	39	21	110	19
Missing	10		3		30	

**Table 2:** QL data collected

	**High dose**			**Conventional**			**All**		
**Time point**	**Expected**	**Returned**	**%**	**Expected**	**Returned**	**%**	**Expected**	**Returned**	**%**
Baseline	198	155	78	192	147	77	390	302	77
6 months	194	144	74	185	141	76	379	285	75
1 year	180	136	76	174	124	71	354	260	74
2 years	145	97	68	144	107	74	289	204	71
3 years	124	65	52	128	73	58	252	138	55
4 years	109	57	52	118	64	54	227	121	53
5 years	105	45	44	112	56	52	217	101	50

**Table 3:** SAQ activity and dimensions scores

	**Sexually active %**	*P* *	**Pleasure score**	*P1* **	*P2* ***	**Discomfort score**	***P1***	***P2***	**Habit**	***P1***	***P2***
Baseline											
All patients	70		13			0	1				
6 months											
High dose	48	<0.001	9	0.55	<0.001	2	0.23	<0.001	0	0.53	<0.001
Conv	62	0.24	9			1			0		
12 months											
High dose	62	0.03	11	0.91	0.03	1	0.63	<0.001	0	0.74	0.11
Conv	81	0.45	11			1			1		
24 months											
High dose	64	0.8	10	na	0.04	1	na	<0.001	1	na	1
Conv	64	1	11			1			1		
36 months											
High dose	70	0.29	9	na	0.02	1	na	<0.001	1	na	0.08
Conv	69	1	11			1			1		
48 months											
High dose	65	0.13	10	na	0.003	2	na	<0.001	1	na	0.49
Conv	55	0.58	9			1			1		
60 months											
High dose	68	0.63	8	na	0.001	2	na	<0.001	1	na	0.45
Conv	65	0.69	9			1			1		

* *P* for sexually active: change since baseline per group: McNemar's test.

** P1 for dimension scores: change since baseline difference between groups: Mann–Whitney test.

*** P2 for dimension scores: overall change since baseline: Mann–Whitney test.
